# Deep learning-based automated lesion segmentation on mouse stroke magnetic resonance images

**DOI:** 10.1038/s41598-023-39826-8

**Published:** 2023-08-16

**Authors:** Jeehye An, Leo Wendt, Georg Wiese, Tom Herold, Norman Rzepka, Susanne Mueller, Stefan Paul Koch, Christian J. Hoffmann, Christoph Harms, Philipp Boehm-Sturm

**Affiliations:** 1 Department of Experimental Neurology and Center for Stroke Research, Charité-Universitätsmedizin Berlin, corporate member of Freie Universität Berlin and Humboldt-Universität zu Berlin, Berlin, Germany; 2 Charité-Universitätsmedizin Berlin, NeuroCure Cluster of Excellence and Charité Core Facility 7T Experimental MRIs, Berlin, Germany; 3 Scalable Minds GmbH, Potsdam, Germany; 4 Berlin Institute of Health (BIH), Berlin, Germany; 5 German Center for Cardiovascular Research (DZHK), Berlin, Germany; 6 Einstein Center for Neuroscience, Berlin, Germany; 7 NeuroCure Clinical Research Center, Charité-Universitätsmedizin Berlin, Berlin, Germany

**Keywords:** Stroke, Cerebrovascular disorders, Experimental models of disease, Preclinical research, Computational science, Computational neuroscience

## Abstract

Magnetic resonance imaging (MRI) is widely used for ischemic stroke lesion detection in mice. A challenge is that lesion segmentation often relies on manual tracing by trained experts, which is labor-intensive, time-consuming, and prone to inter- and intra-rater variability. Here, we present a fully automated ischemic stroke lesion segmentation method for mouse T2-weighted MRI data. As an end-to-end deep learning approach, the automated lesion segmentation requires very little preprocessing and works directly on the raw MRI scans. We randomly split a large dataset of 382 MRI scans into a subset (n = 293) to train the automated lesion segmentation and a subset (n = 89) to evaluate its performance. We compared Dice coefficients and accuracy of lesion volume against manual segmentation, as well as its performance on an independent dataset from an open repository with different imaging characteristics. The automated lesion segmentation produced segmentation masks with a smooth, compact, and realistic appearance that are in high agreement with manual segmentation. We report dice scores higher than the agreement between two human raters reported in previous studies, highlighting the ability to remove individual human bias and standardize the process across research studies and centers.

## Introduction

In ischemic stroke lesion detection, magnetic resonance imaging (MRI) is frequently used in both clinical and pre-clinical research. T2-weighted (T2w) MR images can be used to detect vasogenic edema and are often used as a proxy of the final infarct. In the hours after an ischemic stroke, the blood-brain barrier is disrupted and leads to fluid building up in the surrounding tissue. The resulting extracellular water increases T2 relaxation time, leading to increased signal in the stroke area. Ischemic brain tissue damage can be observed from T2w MR images as early as 3.5 h after the onset of stroke and its volume continues to increase up to 24 h after stroke^1^. T2w MR images acquired in this phase have become an important outcome parameter in rodent stroke research.

A challenge in stroke MRI research is that lesion segmentation often relies on manual tracing by trained experts. Manual segmentation is labor-intensive, time-consuming, and prone to inter- and intra-rater variability^2–5^. Automated lesion segmentation is therefore crucial for both efficiency and reliability. Deep learning-based methods have been developed for various other segmentation tasks in rodent brain, such as skull-stripping in healthy^6–10^ and injured^11,12^ brain, tissue segmentation^13^, hippocampus segmentation^14^, and hemisphere segmentation^13^, relieving the effect of human bias and accelerating the process. Automated lesion segmentation is essential for developing a fully automated processing of rodent stroke MR images. Atlas registration in the presence of a stroke lesion is challenging, and we have previously shown that this can only be effectively done if an algorithm is informed with a lesion mask^15^.

Several automated lesion segmentation algorithms have been developed for human data^16–18^ (for reviews, see^19–21^). Meanwhile, such tools and methods for preclinical data are lagging. This poses a problem since methods developed for human data are often not transferable to animal data. Plus, preclinical studies often entail large numbers of MR scans or multi-center datasets, creating a bottleneck for data analysis pipelines. There are only a few (semi-) automated methods developed for rodent data. Jacobs et al.^22^ developed an unsupervised lesion segmentation model for rat data with an iterative self-organizing data analysis technique (ISODATA), a K-means derived clustering technique. A multiparametric ISODATA was applied to MR data to characterize ischemic lesion tissue in rats. Ghosh et al.^23,24^ developed an automated ischemic lesion segmentation algorithm for neonatal rat brains using a hierarchical recursive region splitting (HRS) approach. Mulder et al.^2^ developed a level-set-based lesion segmentation algorithm for mouse data that requires one T2 MR sequence as input. Castaneda-Vega et al.^25^ trained a random forest classifier for ischemic stroke lesion segmentation in rat brains. However, most of these methods require some degree of manual intervention, require different parameters for each dataset for optimal performance, and are often dependent on the performance of other steps, such as preprocessing. Valverde et al.^4^ presented RatLesNetv2, a Convolutional neural network (CNN)-based Rodent Brain Lesion Segmentation algorithm for T2w MR images. However, RatLesNetv2 requires users to train their own model using their data.

In this exploratory study, we developed a fully automated ischemic stroke lesion segmentation method for mouse T2w MR images. The automated lesion segmentation builds on top of U-Net^26^, a convolutional neural network commonly used in biomedical image segmentation. We trained and evaluated the automated segmentation method on a large dataset of 382 T2w mouse MR images. Our approach produced realistic segmentations in high agreement with manually segmented lesion masks. This study is among the first U-Net-based methods for mouse brain stroke image segmentation. In addition, the dataset used in this study is openly available on zenodo (https://doi.org/10.5281/zenodo.6379878) and presents one of the largest public T2w mouse stroke lesion repositories to date. This study thus contributes to making preclinical stroke lesion segmentation on MR images more standardized across studies and labs.

## Results

The resulting segmentation masks accurately detected lesions and produced a smooth, human annotator-like appearance. Incidence maps of manually segmented (median volume 26.73 mm$^3$, IQR [7.65–48.27]) and automated (median volume 23.48 mm$^3$, IQR [7.94–47.27]) lesion masks are shown in Fig. 2a. We also calculated false positives and false negatives across all test datasets by subtracting manual lesion masks from automated lesion masks, shown in Fig. 2b. Qualitative inspection of the spatial distribution showed the elevated incidence of false positives at the borders between cortical and subcortical structures and more homogenous distribution of false negatives across the lesion territory (Fig. 2b).

**Figure 1:**
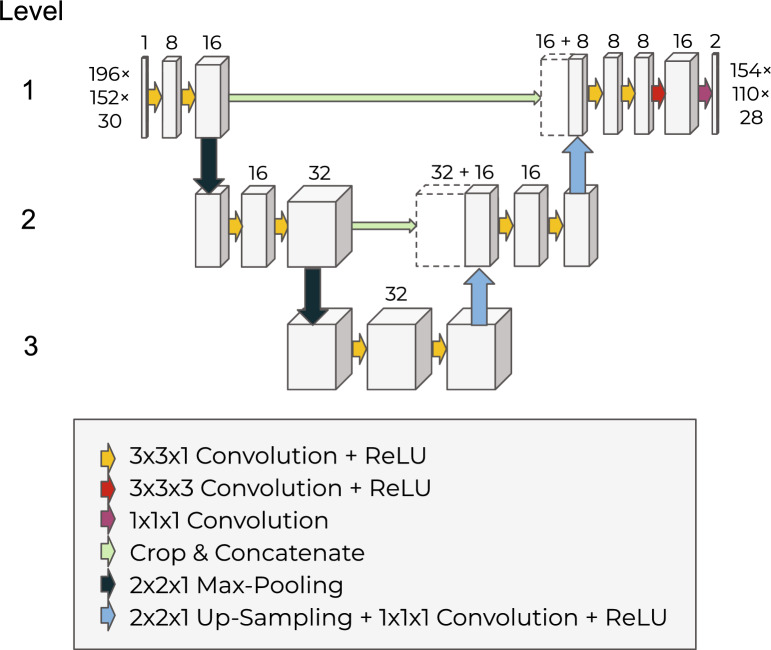
Network architecture and processing pipeline of the automated method.

**Figure 2:**
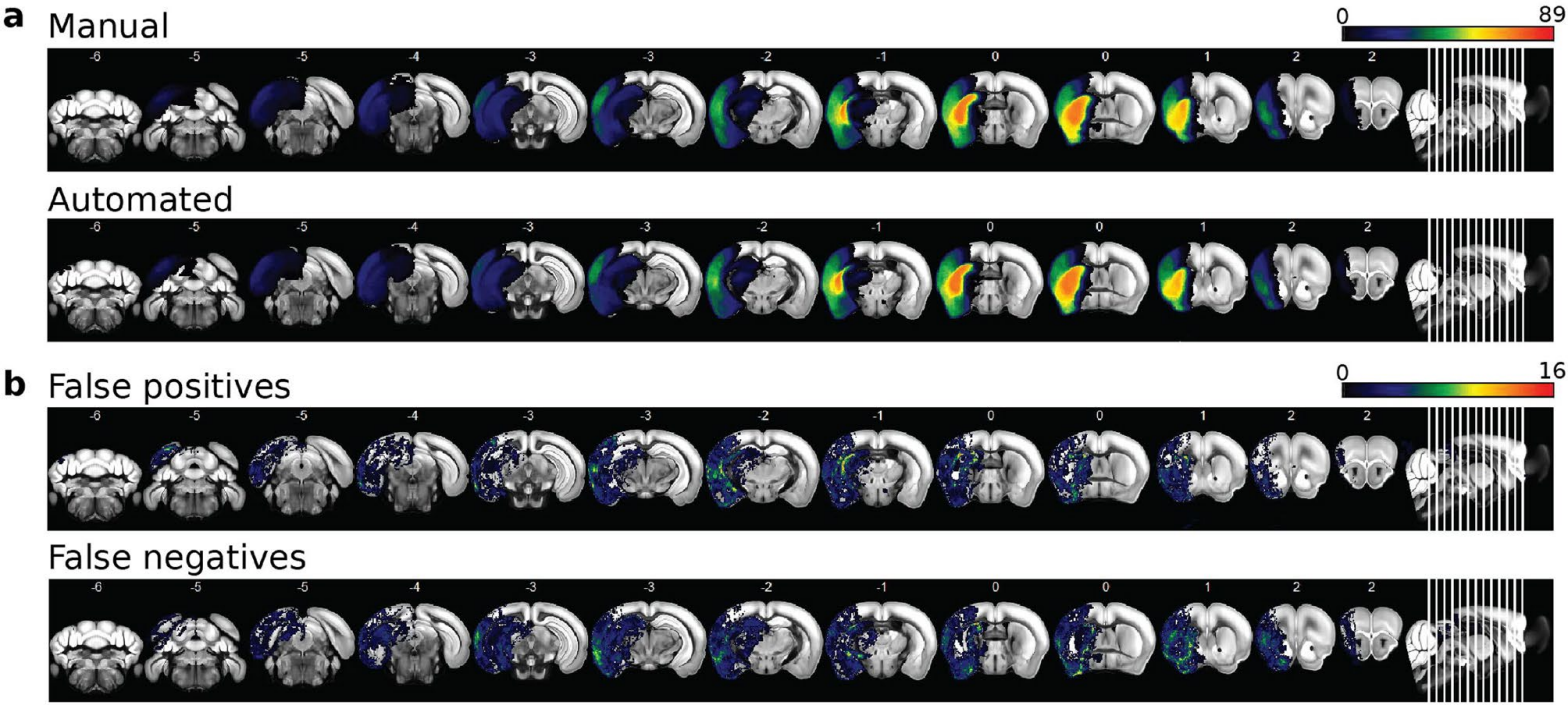
Incidence maps of (**a**) manual and automated lesion masks, and (**b**) false positives and false negatives across all evaluation datasets (n = 89).

The evaluation set included 12 scans of control animals without any lesions. Of those, the automated lesion segmentation detected small lesion masks in 10 datasets, with a median lesion volume of 0.34 mm$^{3}$, IQR [0.19–1.45].

**Figure 3:**
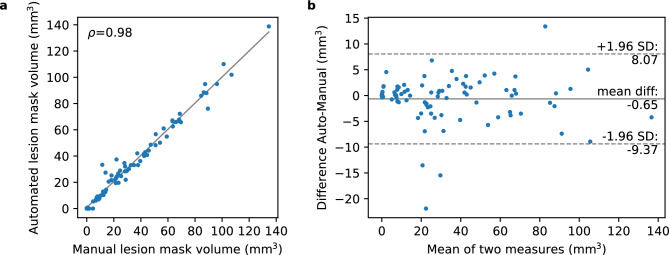
Relationship between the lesion mask volumes of the two methods (**a**) Spearman correlation and (**b**) Bland-Altman plot comparing agreement between the manual and automated lesion mask volumes.

Figure 3a shows the Spearman correlation between manual and automated lesion mask volumes. The plot shows a very high level of correlation, with $\rho$ = .98, n = 89, P = $4.81 \times e^{-61}$. The corresponding Bland-Altman plot (Fig. 3b) indicates very high agreement between the two methods. No large systematic error was found but the automated algorithm tended to underestimate lesion volume for a few of the smaller lesions. Overall, we report a median Dice score of 0.92, IQR [0.86–0.96], a median sensitivity of 0.93, IQR [0.85–0.97], a median specificity of 1.00, IQR [1.00–1.00], and a median precision of 0.93, IQR [0.87–0.97]. In addition, we had an average Dice score of 0.89 ± 0.12.

**Figure 4:**
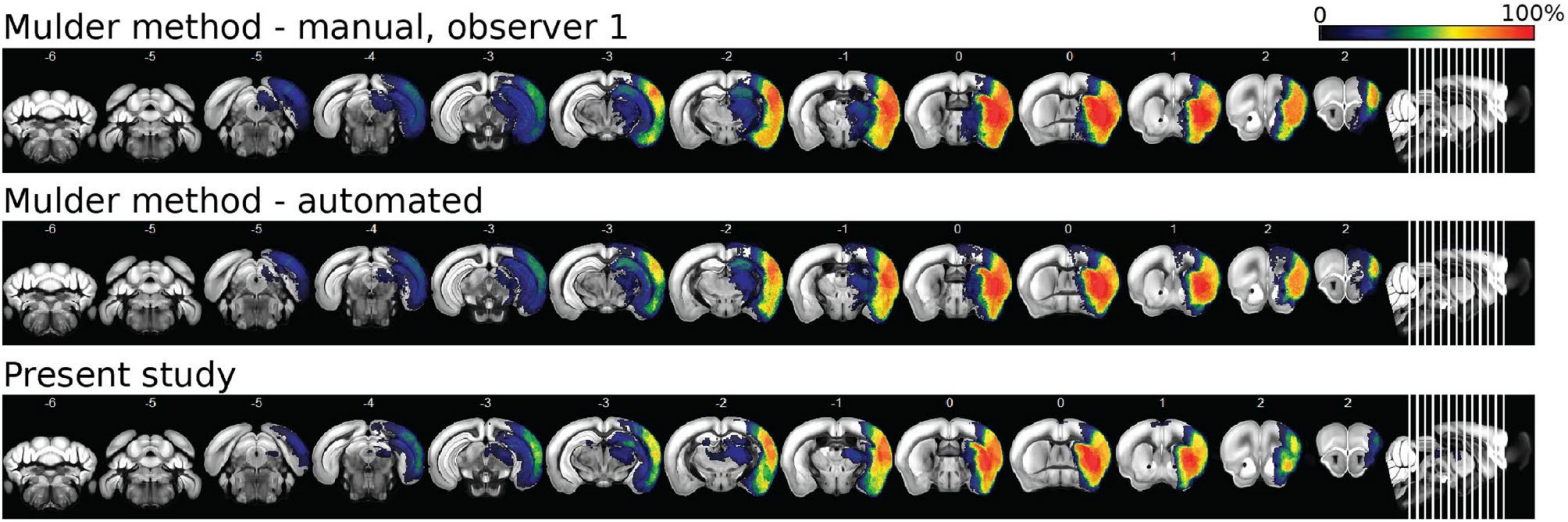
Incidence maps of automated lesion masks generated by Mulder et al.^2^ and by the present study.

To test the generalizability of our automated lesion segmentation method on other datasets, we evaluated our approach on an independent dataset^27^ and compared it with the performance of their method (see Section “Methods” for more details). The results are shown in Table 1. Overall, the automated segmentation was able to segment the lesions reliably and with high accuracy. Our lesion masks tended to be smaller and more compact than lesion masks from their method, as shown in Fig. 4. We had an average Dice score of 0.76 compared to 0.86 of Mulder et al.^2^.

**Table 1:** Comparison of performances of automated lesion segmentation algorithm by Mulder et al.^27^ vs. algorithm of the present study on open data repository of Mulder et al.^27^ Observer 1 vs. observer 2 refer to lesion masks produced by each of the two manual tracers from their study.

Age (months)	Time between infarct induction and MRI	Mulder et al. method		Present study	
		Observer 1	Observer 2	Observer 1	Observer 2
3	18 h (n=6)	0.88 ± 0.03	0.89 ± 0.03	0.789 ± 0.05 (0.74 - 0.87)	0.81 ± 0.05 (0.77–0.90)
	4 d (n=3)	0.85 ± 0.01	0.87 ± 0.01	0.68 ± 0.034 (0.63 - 0.72)	0.712 ± 0.03 (0.69–0.75)
1, 12	48 h (n=10)	0.86 ± 0.07	0.85 ± 0.07	0.80 ± 0.06 (0.66 - 0.87)	0.76 ± 0.08 (0.64–0.87)
Average		0.86	0.87	0.76	0.76

The mean dice coefficient across animals within each group and the average of the mean dice coefficient across three groups are shown. For the present study, errors refer to standard deviation. For Mulder et al.,^2^ the type of error was not reported. We additionally report the range of dice scores with parenthesis.

## Discussion

We have presented an automated algorithm for ischemic lesion segmentation from mouse T2w MR images. Our reported median Dice score of 0.92 shows that the automated lesion segmentation produces realistic segmentations that very closely resemble human annotations. In contrast, previous studies showed that annotations made by two human annotators can have a Dice score as low as 0.73 or 0.79^2,4^. In addition to the fact that manual segmentation can require months or years of practice to build expertise, even trained experts can show low intra-rater reproducibility^5^. Moreover, although each manual segmentation took around 5 min per data set, this can quickly add up to hours, especially in preclinical studies with large data sets. This automated method constitutes a crucial step to overcoming human bias, producing reproducible segmentations, and speeding up the process.

There exist a lot more automated stroke lesion segmentation methods developed for human data^19–21^. Likewise, there are more publicly available stroke MR data sets for clinical research^5,18^ compared to preclinical studies. We address this problem of reverse translation by developing an effective automated method for mouse models of stroke and by making our data openly available.

The extent to which the performance of our model could generalize to human stroke data is uncertain. In animal studies of ischemic stroke, artificially introduced lesions are always located in the same, predictable region. This is unlike cases of stroke in humans, where lesions are more complicated and distributed. In addition, MRI for preclinical studies is often much more high-tech and high-resolution compared to data from clinical studies.

The convolutional neural network architecture used in our approach is based on a variation of the 3D U-Net^26^ design, a highly successful segmentation model for bioimaging tasks. As an end-to-end deep learning approach, the automated lesion segmentation requires very little preprocessing and works directly on the raw MRI scans. Unlike some of the previous automated lesion segmentation approaches^2,23^, the present study is fully automated and it is not dependent on other preprocessing steps, such as registration or skull stripping. This is crucial in achieving a fully automated pipeline for processing rodent stroke MR images, especially since we have previously shown that such preprocessing steps are prone to error in presence of a stroke lesion^15^.

Our approach is similar in design to the recently published RatLesNetv2-architecture^4^ developed for rat data. However, we used a much larger training set and incorporated 3D information across the z-axis into our predictions. They report average dice scores (inter-rater: 0.73 ± 0.12, RatLesNetv2: 0.81 ± 0.16) on their evaluation set that are lower than our best-performing evaluation score (average dice score of $0.89 \pm 0.12$).

We further tested the automated lesion segmentation on the dataset provided by Mulder et al.^27^ and tested generalizability across scans from a different center with different imaging characteristics (lower resolution; T2 maps instead of T2w images; different scan times after infarct induction). Our Dice score on Mulder et al.’s data^27^ fell short of their performance (Table 1). However, their ground-truth annotations contain holes, rough and fringed edges, and spots of isolated voxels since a fixed intensity threshold based on contralateral mean and standard deviation was used without further postprocessing. These differ considerably from ground-truth labels in our dataset, which mostly contain compact lesion masks with smooth edges. We defend the use of compact lesion masks based on the following two arguments. First, consistent with literature^4,28^, we argue compact lesion masks are more realistic and comparable to manual annotations since hypoperfusion in large vessel stroke varies smoothly in space and not at the order of single voxels. Second, our manual lesion masks were obtained using a semi-automated manner using a thresholding function and the lesion masks obtained using the same procedure have been validated by comparison to histological data using TTC staining in a previous study^15^, where we report an excellent correlation. Given that TTC shows very little variation on the scale of a single voxel, this further supports our assumption of compact masks being consistent with the ground truth. A general limitation of deep learning-based automated segmentation methods is that the automated segmentations are biased by the manually delineated training set. We thus tested our method on an independent data set, acquired from a different data center, using different imaging methods and modalities. In addition, their manual lesion masks had different characteristics than ours, as discussed above, and our segmentations were compared to their manual lesion masks for evaluation. Despite these differences, our method showed reliable performance when tested on their dataset, highlighting its generalizability.

The comparison between our automated method and other previously published methods is limited to the application of our algorithm to the data published by Mulder et al.^27^. To our knowledge, Mulder et al.^2^ is the only end-to-end approach developed for mice data thus far. We could not test our data using Mulder et al.^2^ method since their algorithm only takes T2 maps as input which were unavailable for our data. This points to another advantage of our automated method, which is that it can take any image in NIfTI format as input, including T2w images and T2 maps, on which the stroke appears hyperintense. Another limitation of the comparison is that our method was only evaluated on 19 independent data sets, which is much smaller than the size of our training and test data sets. We were, unfortunately, not able to find any other openly available mice stroke MR Image data sets we could use.

T2w images acquired at this time point need to be corrected for the effect of brain edema since swelling due to post-stroke edema leads to a significant overestimation of lesion volume compared to histology^25,29^. A study showed that hyperintensity in T2w at approximately 7 h after stroke represents the final lesion volume and that the subsequent increase in the signal would mainly reflect swelling^1^. The correction could be performed using the atlas registration approach by Koch et al.^15^ or the model by Gerriets et al.^29^. With this consideration in mind, T2w images are the most commonly used imaging technique to estimate the final infarct in animal studies of experimental stroke^30^ and and serve as a useful measure for correlation of lesion site with other imaging biomarkers.

Diffusion Weighted Imaging (DWI), in addition to T2w images, is also routinely applied for ischemic stroke segmentation. Whereas T2w images are sensitive to vasogenic edema, apparent diffusion coefficient (ADC) maps derived from DWI indicate cytotoxic edema, i.e. water diffusion restrictions due to cellular swelling. A reduction in ADC is observed in the first few hours after stroke induction and it is shown to be a strong predictor of final lesion volume^31,32^. ADC and T2w data measure partly overlapping and distinct microstructural lesion traits, and lesion segmentation from ADC and T2w show different patterns at 24 hours after stroke^22,25^. In addition, the combined evaluation of ADC and T2w is shown to lead to a more accurate prediction of the final stroke volume^22,25^.

To test whether the method could also generalize across different MR contrasts, we ran the automated method on a preliminary dataset of 5 DWI acquired after permanent distal MCAo in the same mouse model. Here, the automated method showed poor performance, with an average dice score of 0.24. Thus, a limitation of our method is that, although we show that it could be generalized across data sources and small changes in surgery, it may not generalize well across different MR contrasts and surgery procedures.

Another limitation of our approach is that it is only end-to-end if B1+/− is homogenous. Intensity variation due to B1+/− and bias field issues are common in high-field MR images acquired with a preclinical scanner. With the presence of B1+/− inhomogeneity, an extra preprocessing step involving B1 correction, e.g. using ANTs N4 Bias Field correction^33^ or SPM’s bias field correction using simultaneous segmentation/registration procedure^34^, is necessary.

Finally, a limitation is that we did not have ground truth based on histology for this study, and the manual segmentations from MRI served as the “ground truth” for model training. However, we would like to stress again that the manual segmentations obtained through the same method have been validated against histology data in a previous study^15^, where we report high correlation between the lesion volumes.

To conclude, a fully automated end-to-end deep learning approach to segment stroke lesions in preclinical studies has been trained on one of the largest datasets of T2w MR images acquired in subacute murine stroke to date. The method performed well compared to manual segmentation and will help to standardize MRI-based lesion volumetry between studies and labs. Furthermore, it can now be used to inform other algorithms needed for rodent MR image preprocessing that previously relied on manual interaction.

## Methods

### Animals

We retrospectively reanalyzed male C57/BL6 J mice (Janvier, Germany) from previous stroke studies, most of which are unpublished. Animals underwent middle cerebral artery occlusion (MCAo) at the age of 10–12 weeks. N = 331 animals had a detectable lesion on T2w images, n = 51 animals that did not display a lesion (unsuccessful surgery and sham) were included to control for false positive results. Experimenters were blinded to the condition of the animals. Mice were housed in a temperature ($22\pm 2\,^\circ$C), humidity ($55\pm 10\%$), and light (12/12-h light/dark cycle) controlled environment. All surgeries were performed under 1.5–2% isoflurane in a 70% nitrous oxide and 30% oxygen mixture. During surgery, core body temperature was maintained at $37\pm 0.2\,^\circ$C with an automated rectal probe and heat blanket. After surgery, a topical application of 1% bupivaccain gel was applied on surgical wounds. All experiments were approved by the responsible institution (Landesamt für Gesundheit und Soziales under registration numbers G0197/12, G0005/16, G0057/16, G0119/16, G00254/16, G0157/17, and G0343/17) after positive evaluation by the local ethics commission for animal experiments. Studies were performed in accordance with the German Animal Welfare Act and reporting follows the ARRIVE^35^ guidelines.

### MCAo

After anesthesia induction, a midline neck incision was made to expose the left carotid artery. A filament (190 µm diameter, Doccol, Sharon, MA/USA) was introduced into the external carotid artery (ECA) and advanced to the origin of the left MCA via the internal carotid artery. After 45 min, the filament was withdrawn. For sham animals, the filament was advanced to the MCA and withdrawn immediately. Both the common carotid artery and the ECA were ligated and remained occluded during reperfusion^36^. All MCAo were performed by two experienced surgeons.

### MRI measurements

Mice were anesthetized with isoflurane (2–2.5% at initiation and 1–2% during the scanning period) in a 70:30 nitrous oxide:oxygen mixture. Respiration rate was monitored with a pressure-sensitive pad using MRI compatible equipment (Small Animal Instruments, Inc., Stony Brook, NY, USA). T2w images were acquired 24 h post-stroke surgery on a 7 T MR scanner (Bruker, Ettlingen, Germany) and a 20 mm inner diameter transmit/receive quadrature volume coil (RAPID Biomedical, Rimpar, Germany). A 2D RARE sequence was used (repetition time/echo spacing/effective echo time = 4.2 s/12 ms/36 ms, RARE factor = 8, 32 consecutive 0.5 mm thick slices, FOV = (25.6 mm$^2$), image matrix = 256 $\times$ 196 zerofilled to 256 $\times$ 256, 4 averages, scan time 6:43 min).

### Manual lesion segmentation

The manual lesion masks were created in a semi-automated manner by tracing hyperintense lesions using a thresholding function in ANALYZE 10.0 software (AnalyzeDirect, Overland Park, KS, USA). First, an auto-thresholding function was used to get a first impression of the lesion size and location. Next, a trained expert manually segmented the lesion by visual inspection. The lesion masks were of the same dimensionality as the T2w images and were exported in NIfTI format. The manual segmentation was performed by an experienced researcher with over 20 years of experience and whose tracings were validated against histology in previous studies^15^. Each manual segmentation took around 5 min.

### Independent dataset

An independent dataset from a previous study^27^ was used to further evaluate the automated lesion segmentation. This independent dataset is different from that of the present study in that it is of lower resolution (128$\times$128 and 196$\times$196, instead of 256$\times$256), contains quantitative T2 maps (instead of T2w images), and had different scan times after the infarct induction. The authors provide two manual annotations from independent observers, which were segmented by applying various thresholding operations in ImageJ in a semi-automated manner. Some of the low signal-to-noise ratio data in the dataset, therefore, contained very fragmented lesion masks with small holes (order of one or few voxels). We evaluated the automated lesion segmentation on a subset of 19 higher-quality datasets (Cologne-Set 1, Cologne-Set 2), which we found to have lesion masks that were less fragmented, and thus more realistic, than other datasets.

### Automated lesion segmentation

#### Network architecture

In order to automatically segment the lesion, a variant of the popular U-Net was trained^26^. The architecture follows an encoder-decoder design but adds “skip connections” between intermediate layers of the same spatial resolution.

We cropped the images to speed up the training process (less pixels to predict) and to avoid a strong influence of black-only pixels on the distribution of the input values. Since the image field-of-view was placed concentric with respect to the mouse head, the network input of 196$\times$152$\times$30 still contained all tissue and the skull around the brain giving the model all the available “texture” for training.

We trained and evaluated a number of different hyperparameter configurations and only report the best-performing setting in our study. Amongst others, we varied the number of feature maps per layer, the kernel size of the convolutional layers, the depth of the U-Net (=number of layers), and tried constant and decaying learning rate schedules. We had to strike a balance with the layer and feature count to avoid overfitting on the limited training dataset. Given the volumetric data from MRI scans, we experimented with different configurations of 2D and 3D convolutional layers to take z-context into account. Due to the significantly higher spatial image resolution along the X- and Y-axes compared to the z-axis this required a good balance. To aid with generalizability over multiple datasets both from within our own collection of training data and with respect to other open source data, we experimented with various data augmentation techniques. In addition to scaling, rotating, and mirroring the training data, we added various amounts of artificial noise, blur, brightness- and contrast manipulations.

As shown in Fig. 1, two max-pooling layers were applied, allowing the network to analyze the image at three distinct spatial resolutions. The upsampling was implemented as nearest-neighbor upsampling, followed by a convolutional layer with kernel size 1$\times$1$\times$1 and ReLU activation. Between consecutive downsampling or upsampling steps, two convolutions with kernel size 3$\times$3$\times$1 and ReLU activation were applied. At last, a 3$\times$3$\times$3 convolution with ReLU activation was applied, followed by a 1$\times$1$\times$1 convolution with a softmax activation, the output of which is interpreted as the background and lesion probabilities. All in all, the network maps a 196$\times$152$\times$30 image tensor to a 154$\times$110$\times$28$\times$2 tensor of probabilities. The receptive field of the network is 43 $\times$ 43$\times$3 voxels which equals 4.3 $\times$ 4.3$\times$1.5 mm$^3$.

The number of feature maps was set to 8, 16, and 32, depending on the level of the U-Net. The number of feature maps of the next level was already used on the last convolution before the down-sampling^37^. Batch normalization layers were purposefully excluded because we experienced issues in low-contrast parts of the dataset when batch normalization was included. The network had 42,442 trainable parameters.

#### Model training

The boxes were split randomly into a training (n = 293) and test set (n = 89). After training, the test set was used to evaluate the performance of the finalized model. The (0, 255) interval of intensity values was scaled to the ($-1$, 1) interval by transforming them via I := (I - 127.5) / 127. As a data augmentation, voxel-wise Gaussian noise was added to the training samples with a mean of 0 and a standard deviation of 0.45.

The voxel-wise loss was computed as the categorical cross-entropy between the predicted probabilities and the one-hot encoded ground truth class. The training loss was defined as the total categorical cross-entropy across all voxels in a batch of eight training examples. Voxels were weighted inversely proportional to the frequency of the respective class.

All kernels were initialized according to Glorot and Bengio^38^. Biases were initially set to zero. The network was trained to minimize the training loss using the Adam optimizer^39^ with a learning rate of 1e-4 and a batch size of 8.

#### Implementation

The automated lesion segmentation method was implemented in TensorFlow and Python using the Voxelytics framework (https://voxelytics.com; scalable minds, Potsdam, Germany). Training and predictions were executed on a system with an NVidia Geforce GTX1080, an Intel Core i7-6700 CPU @ 3.40GHz, and 64GB RAM.

### Qualitative comparison and Statistical Analysis

To create incidence maps, T2w images and corresponding lesion masks were registered to the Allen mouse brain atlas using ANTx2 as described previously (https://github.com/ChariteExpMri/antx2)^15^. Incidence maps were generated by displaying voxel-wise mean of lesion masks across animals (expressed in percent) in atlas space. For each animal, a map of false positives was generated by voxel-wise subtraction of the manually generated lesion mask from the automatically generated lesion mask in atlas space followed by removal of all values< 1. Incidence maps of false positives were then generated by voxel-wise mean of false positive maps across animals (expressed in percent). For incidence maps of false negatives, the same procedure was performed except the automatically generated mask was subtracted from the manually generated mask. For other evaluation metrics described below, we used images and lesion masks in their native space.

We evaluated the automated lesion segmentation on a dedicated test set consisting of 89 datasets. We used several metrics for assessment. First, we used total lesion volume, which is one of the most important readout parameters in preclinical stroke studies. Since the lesion volumes are not normally distributed, the manual and automated lesion volumes were compared using Spearman’s rank correlation and Bland-Altman analyses. Correlation and Bland-Altman analyses and visualization were performed using Python (version 3.8.8). Secondly, we used the Dice coefficient^40^, which measures the overlap volume between two regions and is popularly used for performance evaluation in the field of image segmentation. In addition, we report sensitivity, specificity, and precision rate. Sensitivity evaluates the capability to correctly detect true positives, whereas sensitivity focuses on the correct detection of true negative voxels. Precision is a measure of reliability that evaluates the fraction of predicted positives that is actually positive. As these measures are not normally distributed we report them with median and interquartile range (IQR). For comparison with previous studies that report only the average dice score, we also report the average dice score ± standard deviation.

## Data Availability

The datasets used for this study can be found in the Zenodo repository, https://doi.org/10.5281/zenodo.6379878.

## Code availability

The model and example Python code for automated segmentation is made available in this GitHub repository, https://github.com/scalableminds/stroke-lesion-segmentation. The source code for ANTx2 used for T2w image registration for incidence maps is available in this GitHub repository, https://github.com/ChariteExpMri/antx2.
